# Tumor Characteristics Associated with Lymph Node Metastasis and Prognosis in Patients with ERBB2-Positive Gastric Cancer

**DOI:** 10.1155/2022/7592046

**Published:** 2022-08-25

**Authors:** Ran Xu, Yisheng Zhang, Jun Zhao, Ke Chen, Zhengguang Wang

**Affiliations:** ^1^Department of General Surgery, The First Affiliated Hospital of Anhui Medical University, Hefei, Anhui, China; ^2^Department of General Surgery, The Yijishan Hospital of Wannan Medical College, Wuhu, Anhui, China

## Abstract

Gastric cancers (GCs) that express human erb-b2 receptor tyrosine kinase 2 (ERBB2, also known as HER2) account for 7.3%–20.2% of GCs. The pathological and prognostic factors associated with lymph node metastasis of such tumors are still unclear. Therefore, we aimed to identify the risk factors for lymph node metastasis and prognostic factors of patients with ERBB2-positive GC. We conducted a retrospective analysis of pathological specimens after D2 radical surgery for locally advanced GC and D1+ surgery performed for early GC in our hospital from January 2015 to December 2018. Patients with ERBB2-positive GC were selected and the potential risk factors for lymph node metastasis and potential factors affecting prognosis were evaluated. Among 1,124 GC patients, 122 diagnosed with ERBB2-positive GC were included in the study. We found that risk factors for lymph node metastasis included tumor size (hazard ratio (HR)- 6.213, 95% confidence interval (CI)- 2.097–18.407, *p* = 0.001), neural invasion (HR- 2.876, 95% CI - 1.011–8.184, *p* = 0.048), and vascular invasion (HR- 16.881, 95% CI - 5.207–54.727, *p* < 0.001). T stage (HR- 4.615, 95% CI - 2.182–9.759, *p* < 0.001) and vascular invasion (HR- 3.036, 95% CI - 1.369–6.736, *p* = 0.006) were significant prognostic variables. These findings shed new light on the pathology and prognosis of patients with ERBB2-positive GC.

## 1. Introduction

Gastric cancer (GC) is a highly fatal disease that has attracted extensive public attention. GC is the fifth most commonly occurring cancer, with more than 1.08 million new cases in 2020 worldwide. In China, the incidence of GC (∼47/100,000) is much higher than in any other region (North America, Northern Europe, and so on) [1, 2]. With the advancement in integrated treatment strategies, the survival rate of patients with GC has improved. Nevertheless, GC is the fourth most common cause of tumor-related deaths [1]. Despite significant progress in early cancer screening, surgical techniques, and postoperative adjuvant chemotherapy, the 5-year survival rate of patients with advanced GC is 10–30% [3, 4]. Since targeted therapy has shown good efficacy in ERBB2-positive breast cancer, scientists have conducted several studies to determine whether targeted therapy has similar efficacy in GC [5–7]. According to the To-GA clinical trial report, targeted therapy can improve the prognosis of ERBB2-positive GC patients [6]. These findings highlight the importance of evaluating the significance of erb-b2 receptor tyrosine kinase 2 (ERBB2, also known as HER2). The consensus criteria for diagnosing ERBB2-positive GC involve the detection of ERBB2 using immunohistochemistry (IHC) scored as IHC grade 3+ or IHC grade 2+ combined with the detection of ERBB2 amplification using fluorescence in situ hybridization (FISH) [7]. Notably, patients with these unique phenotypic subtypes, accounting for 7.3–20.2% of GC, require different treatment strategies [8, 9]. Most studies demonstrate a poor prognosis for ERBB2-positive GC patients, particularly patients with associated clinical features such as serosa invasion, lymph node metastasis, and distant metastasis [10–12]. Contradictorily, some studies have shown no significant correlation between the ERBB2 status and prognosis [13, 14]. Other studies have reported ERBB2-positivity was associated with a poor prognosis in patients with stage-I GC but not with advanced GC [15, 16]. To our knowledge, the risk factors for lymph node metastasis and prognostic factors of ERBB2-positive patients with GC are still not completely understood. Therefore, we aim to investigate the clinical significance of these aspects of ERBB2-positive GC in detail.

## 2. Materials and Methods

### 2.1. Patients

**Figure 1:**
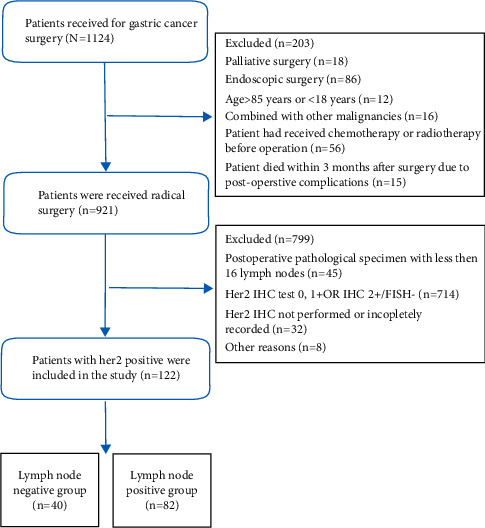
The patient selection strategy.

Records of patients with GC who underwent surgery at the Yijishan Hospital of Wannan Medical College from January 2015 to December 2018 were retrospectively analyzed. Patients' data were included in the study based on the following inclusion criteria: (1) postoperative pathologically confirmed gastric adenocarcinoma; (2) postoperative tumor tissues were analyzed to detect ERBB2 expression (IHC3+, IHC2+, and FISH+; see next section); (3) availability of complete medical records; and (4) availability of complete and valid follow-up information. The exclusion criteria were as follows: (1) age <18 years or > 85 years; (2) synchronous malignancies (secondary excluded); (3) previous gastric malignancies; (4) administration of chemotherapy or radiotherapy before surgery; and (5) death within 3 months after surgery caused by postoperative complications. Patients' baseline and clinicopathological characteristics were obtained through a review of medical records. The disease stage was assigned according to the guidelines of the American Joint Committee on Cancer (AJCC) Tumor-Node-Metastasis (TNM), 7th edition [17]. The inclusion strategy of patients was presented in Figure 1. Patients' data included the following variables: age, gender, histological types of gastric adenocarcinomas, tumor size, tumor location, T stage, N stage, presence or absence of neural invasion, vascular invasion, Lauren type, tumor deposits, surgical procedures, and postoperative adjuvant therapy. All patients provided informed consent before undergoing gastroscopy, surgery, or chemotherapy. The study was reviewed and approved by the Ethics Committee of the Yijishan Hospital of Wannan Medical College (approval number: 2021–083).

### 2.2. Immunohistochemistry (IHC) and Fluorescence In Situ Hybridization (FISH)

**Figure 2:**
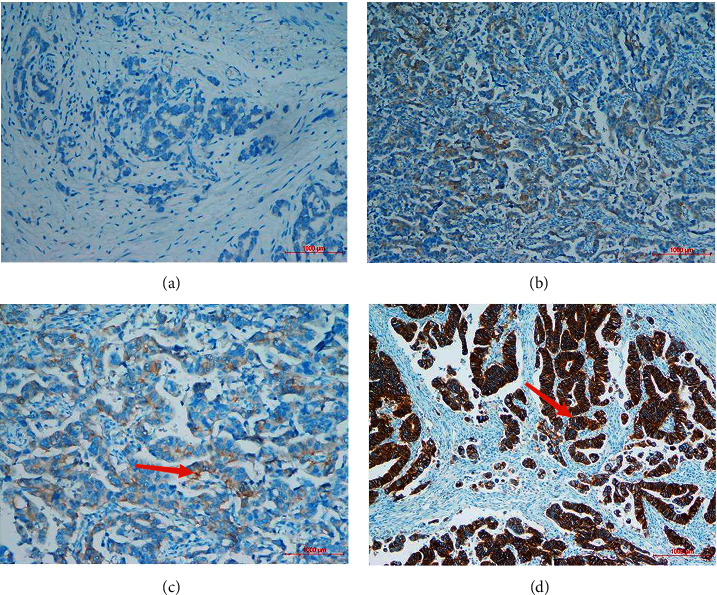
IHC analysis of the ERBB2 expression in GC cells. (a) IHC (0), (b) IHC (1+), (c) IHC (2+), and (d) IHC (3+). Magnification ×200; red arrow indicates ERBB+.

**Figure 3:**
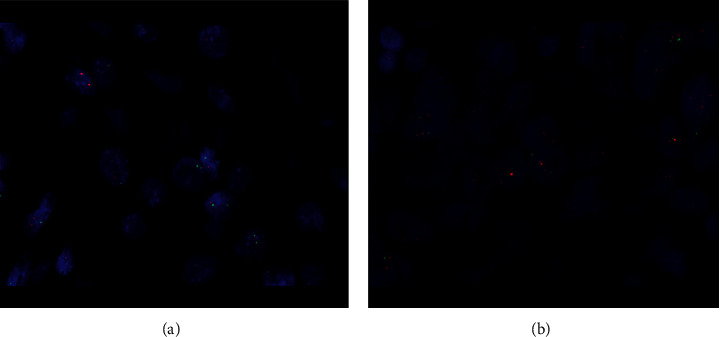
FISH analysis of the ERBB2 amplification. (a) FISH (−) and (b) FISH (+) Magnification ×1,000.

The guidelines for ERBB2 detection in GC recommend adopting a detection strategy combining IHC and FISH [18]. Postoperative GC specimens were embedded in paraffin, and conventional 4 *µ*m consecutive sections were stained with hematoxylin-eosin (HE). IHC and FISH were performed using sections not stained with HE. Anti-HER2/neu (4B5, Roche) monoclonal primary antibody was used to stain ERBB2 using an automated Roche Benchmark GX IHC/ISH system. This antibody was detected at the cell membrane of tumor cells. IHC staining was graded as 0, 1+, 2+, and 3+. IHC0 indicated undetectable or <10% staining of the tumor cell membrane. IHC1+ corresponded to ≥10% of tumor cells exhibiting weak or partially visible membrane staining. IHC2+ corresponded to ≥10% weak to moderate membrane staining of ≥10% of tumor cells. IHC3+ corresponded to strong staining of the basal lateral membrane, lateral membrane, or entire membrane of ≥10% of tumor cells. Furthermore, FISH was also performed on samples with IHC grade 2+. FISH was performed using a Vysis LSI IGH/MAF DF FISH Probe Kit (Abbott Molecular Inc., Des Plaines, IL, USA) using an ERBB2 probe and the hybrid probe for chromosome 17 (CEP17) [19]. After hybridization, the signal counts of ERBB2 and CEP17 were calculated separately and a ratio between them was taken. The FISH results were represented as the intensity ratio between ERBB2 and the chromosome 17 centromere (CEP17) in tumor cells in the highest region of gene amplification and ≥20 consecutive tumor nuclei. A score of more than 2.2 was considered positive. Cases with IHC 3+ or IHC 2+/FISH+ (Figures 2 and 3) were considered ERBB2-positive, while IHC0, 1+, or IHC 2+/FISH–were considered ERBB2-negative [7].

### 2.3. Follow-Up

Patients were followed up through phone or outpatient consultations every three months for a year, every six months for two years after that, and then yearly until death. Overall survival (OS) was defined as the interval between the date of the surgery and the date of the last follow-up. Outpatient examinations include physical examinations, laboratory tests (routine blood tests, blood biochemistry, and analyses of tumor markers such as CEA and CA199) every three months; CT scans every six months, and annual gastroscopy. The median duration of follow-up was 28 months (8–54 months). The last follow-up was conducted on October 31, 2021.

### 2.4. Statistical Analysis

Statistical analyses were performed using SPSS 20.0, and *p* < 0.05 were considered a significant difference between the datasets. Mean ± standard deviation or median ± interquartile range was used to represent the continuous variables, and frequency (%) was used to represent the categorical variables. The Chi-squared or Fisher's exact test was used to compare categorical variables. OS curves were generated using the Kaplan–Meier method, and the log-rank test assessed the differences between the survival curves. The relevant factors for OS were identified using univariate analysis. Variables with *p* < 0.05 in the univariate test were entered into the multivariate Cox regression model to verify the independent risk factors.

## 3. Results

### 3.1. Patients' Characteristics

We identified 122 ERBB2-positive GC patients using the patient-selection strategy). To identify risk factors for lymph node metastasis, the included cases were classified as lymph node-positive (*n* = 82) or lymph node-negative (*n* = 40).

Patients' detailed basic information, pathological data, and relevant clinical data are presented in Table 1. 98 out of 122 patients examined were men. The median age of the patients taken for the study was 69 years (range 33–85 years) with a median tumor size of 4 cm (range 1.2–10 cm). Of the N stage, 40 (32.8%) were classified as pN0, 28 (23.0%) as pN1, 17 (13.9%) as pN2, and 37 (30.3%) as pN3. Of the *T* stage, 15 (12.3%) were classified as pT1, 9 (7.4%) as pT2, 57 (46.7%) as pT3, and 41 (33.6%) as pT4. Primary tumors were classified as moderately differentiated (*n* = 58), moderate to poorly differentiated (*n* = 55), or poorly differentiated (*n* = 10). Patients' tumors were histologically classified as adenocarcinoma (*n* = 101), mucinous adenocarcinoma (*n* = 10), papillary carcinoma (*n* = 4), signet-ring cell carcinoma (*n* = 6), and adenosquamous carcinoma (*n* = 1). Tumors were located in the upper (*n* = 41), middle (*n* = 10), lower (*n* = 58), or entire (*n* = 13) stomach. Gastrectomy approaches included proximal (*n* = 20), distal (*n* = 58), and total (*n* = 44). Most patients were postoperatively administered chemotherapy (*n* = 105). Lauren types were intestinal (*n* = 108), diffuse (*n* = 7), and mixed (*n* = 7). Tumor deposits were present in 21 patients.

### 3.2. Risk Factors for Lymph Node Metastasis in ERBB2-Positive GC Patients

Univariate analyses revealed that tumor size, T stage, vascular invasion, neural invasion, and tumor deposits were risk factors for developing nodal metastases (Table 2). Multivariate analyses revealed that a tumor size >4 cm, vascular invasion, and neural invasion were independent factors for lymph node metastasis (Table 3).

### 3.3. Survival Analysis of ERBB2-Positive Gastric Cancer

**Figure 4:**
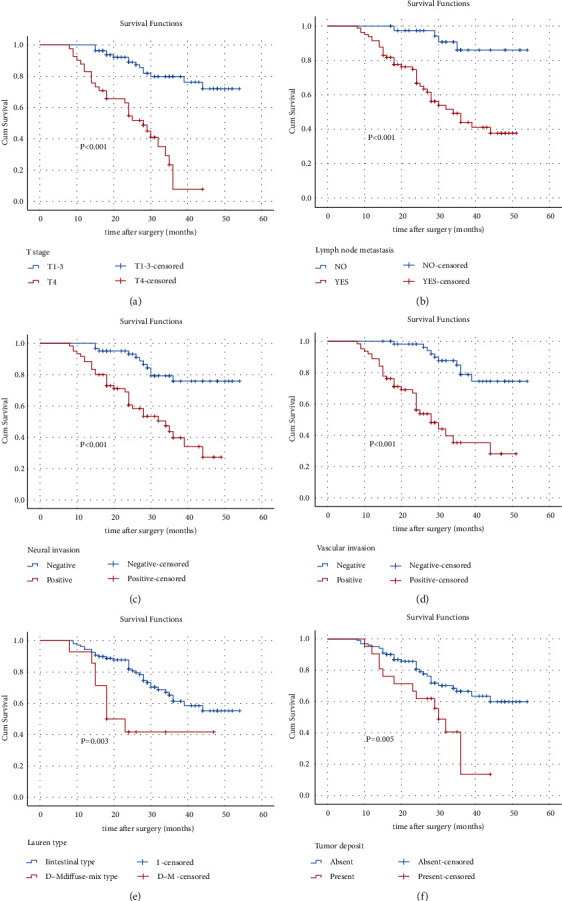
Analysis of overall survival. (a) Overall survival (OS) according to the T stage (T1-3/T4). (b) OS of patients according to lymph node metastasis (no/yes). (c) OS according to neural invasion (negative/positive). (d) OS according to vascular invasion (negative/positive). (e) OS according to Lauren type (I/D–M). (f) OS according to tumor deposit (absent/present).

The 3-year survival rate of patients with ERBB2-positive lymph node metastasis was 48.2%, compared with 86.0% for those without lymph node metastasis (*p* < 0.001). Univariate analysis revealed that T stage, lymph node metastasis, neural invasion, vascular invasion, Lauren type, and tumor deposits were significantly associated with prognosis (Figure 4(a)–4(f)). Multivariate Cox proportional hazards analysis identified T stage (HR- 4.615, 95% CI - 2.182–9.759, *p* < 0.001) and vascular invasion (HR- 3.036, 95% CI - 1.369–6.736, *p*=0.006) as independent prognostic factors (Table 4).

## 4. Discussion

This study analyzed the clinicopathological characteristics and prognosis of patients with ERBB2-positive GC. We found that tumor size, neural invasion, and vascular invasion were risk factors for lymph node metastasis. Further analysis showed that T stage and vascular invasion were factors significantly associated with the prognosis of these patients.

The analysis of the ERBB2 expression in GC is utilized for patients with advanced GC. Patients with ERBB2-positive GC benefit from trastuzumab treatment compared with conventional chemotherapy alone [6]. Multiple studies have analyzed the relationship between ERBB2 positivity and clinicopathological factors in GC and explored the relationship between the ERBB2 status and prognosis [9, 13, 14, 16]. However, there is no consensus regarding the significance of the ERBB2 expression in predicting the prognosis of GC. To our knowledge, the clinicopathological characteristics and prognostic risks of ERBB2-positive GC patients are still unclear.

We analyzed 122 patients with ERBB2-positive GC. The male-to-female ratio was 4.08 : 1, similar to that of GC in Asia [1]. Studies have reported a lower incidence of GC in women than men, which might be related to estrogen in female patients [20, 21]. Research on the factors of lymph node metastasis in GC has been a hot topic [22–24]. However, the factors associated with lymph node metastasis in ERBB2-positive GC patients are unknown. To address this gap in our knowledge, we compared the characteristics of such patients with or without nodal metastasis. We found that a tumor size >4 cm, vascular invasion, and neural invasion were more common in patients with nodal metastases. These findings suggest that patients with one of these risk factors should be considered candidates for lymph node dissection. In clinical practice, the lymph node metastasis of GC plays a crucial role in choosing subsequent treatment, especially for patients with early GC. ERBB-2 positivity has been shown as a high-risk factor for lymph node metastasis in patients with early GC [25]. In this study, the rate of lymph node metastasis in ERBB-2 positive patients was 67.2% (82/122), which is significantly higher than that in ERBB-2 negative patients, which was 48.4% (346/714). Lymph node metastasis was associated with a poor prognosis with univariate analysis but not with multivariate analysis. However, in our experience, the latter finding does not reflect clinical outcomes and might result from a small sample size. Thus, further studies are required to resolve this apparent discrepancy.

Most patients with GC harbor advanced tumors at the time of diagnosis and show a poor prognosis. Our study population (*n* = 122) included 17 patients with stage-I GC and 105 with stages II-IV GC. Survival analysis identified T4 stage, lymph node metastasis, neural invasion, vascular invasion, Lauren type (diffuse-mixed), and tumor deposits as variables significantly associated with a poor prognosis. T stage accurately predicts patients' prognoses with different histological subsets of GC [17]. Here, we found that the 3-year OS of patients with stage T4 (7.8%) was significantly poorer than those with stage T1-3 (79.8%). Furthermore, multivariate analysis showed that the T4 stage was an independent risk factor for the prognosis of this subgroup of patients. Previous studies have shown that GC patients with combined neural and vascular invasion have a poor prognosis [26–28]. This study's univariate analysis suggested that neural invasion and vascular invasion were significant risk factors affecting the prognosis, although multivariate analysis identified only vascular invasion as significant. Nevertheless, these findings indicate that neural and vascular invasion contribute to a poor prognosis. Therefore, in clinical practice, close attention should be paid to the neural and vascular status to help predict outcomes and manage treatment.

The Lauren type is related to the prognosis of patients with GC. For example, evidence indicates that high levels of the ERBB2 expression are associated with the intestinal type, and such patients have a better prognosis than those with the mixed type [13]. Furthermore, according to the ERBB2 status and Lauren classification, the prognosis of patients with GC shows that ERBB2-negative patients with the intestinal type have a better prognosis than those with the ERBB2-positive diffuse type [16]. These findings are consistent with the present study's demonstration that 3-year OS rates were 61.3% and 41.7% of patients with the intestinal or diffuse-mixed types, respectively.

Tumor deposits are associated with the prognosis of patients with GC. Previous studies show that tumor deposits in patients with GC indicate an aggressive malignant phenotype with a poorer prognosis [29, 30]. Our findings suggest that patients with tumor deposits experienced a significantly shorter survival than those without, although tumor deposits were not identified as an independent risk factor for prognosis.

Data indicating that tumor size influences the prognosis of GC is controversial [31–33]. Our present study shows that 3-year OS rates were 66.3% and 49.7% of patients with tumors ≤4 cm and >4 cm, respectively, although the difference is not statistically significant. We believe that as the tumor grows and becomes larger, the later the tumor staging, the worse the patient's prognosis, leading to inconsistent results, which might be related to the tumor size defining the grouping.

Chemotherapy is an effective treatment for advanced GC, which prolongs survival and improves the quality of life [34, 35]. A recent study shows that SOX plus trastuzumab is safe and effective for treating advanced ERBB2-positive GC [36]. Our present study shows that patients in the CapeOX/SOX group experienced higher 3-year survival rates than patients in the S-1 group, although the difference was not statistically significant. This finding may explain the inconsistent staging of the baseline pathology of the two groups. Unfortunately, only six patients who developed recurrence after surgery underwent trastuzumab therapy. Subgroup analysis was not possible because of the low number of eligible patients and their inconsistent baseline characteristics. Therefore, further research is required to confirm and extend these findings.

The limitations of the present study are as follows: 1. ERBB2-positive GC is rare, and therefore, the number of patients included here was relatively small. 2. Selection bias is inherent in retrospective studies such as this. 3. Data on postoperative targeted therapy were incomplete, mainly because most patients could not afford trastuzumab treatment.

## 5. Conclusions

Our study demonstrates that tumor size, neural invasion, and vascular invasion were significantly associated with node metastases in ERBB2-positive GC patients. Furthermore, T stage and vascular invasion served as independent prognostic variables. These new findings might contribute toward optimizing treatment and guide efforts to identify novel therapeutic targets for this deadly subtype of GC.

## Figures and Tables

**Table 1:** Patients' characteristics.

Variable	*N* (%)
*Gender*	
Male	98 (80.3%)
Female	24 (19.7%)

*Age (years)*	
≤60	20 (16.4%)
>60	102 (83.6%)

*Tumor size*	
≤4 cm	64 (52.5%)
>4 cm	58 (47.5%)

*AJCC T stage*	
PT1	15 (12.3%)
PT2	9 (7.4%)
PT3	57 (46.7%)
PT4a/PT4b	41 (33.6%)

*AJCC N stage*	
PN0	40 (32.8%)
PN1	28 (23.0%)
PN2	17 (13.9%)
PN3	37 (30.3%)

*AJCC stage*	
I	17 (13.9%)
II	48 (39.3%)
III	55 (45.1%)
IV	2 (1.6%)

*Histologic classification*	
Moderately differentiated	58 (47.5%)
Moderately poorly differentiated	54 (44.3%)
Poorly differentiated	10 (8.2%)

*Histology*	
Conventional AD	101 (82.8%)
Mucinous AD	10 (8.2%)
Papillary AD	4 (3.3%)
Signet-ring cell carcinoma	6 (4.9%)
Adenosquamous carcinoma	1 (0.8%)

*Tumor location*	
Upper 1/3	41 (33.6%)
Middle 1/3	10 (8.2%)
Lower 1/3	58 (47.5%)
Mix	13 (10.7%)

*Vascular invasion*	
(−)	59 (48.4%)
(+)	63 (51.6%)

*Neural invasion*	
(−)	62 (50.8%)
(+)	60 (49.2%)

*Type of gastrectomy*	
Proximal subtotal gastrectomy	20 (16.4%)
Distal gastrectomy	58 (47.5%)
Total gastrectomy	44 (36.1%)

*Postoperative chemotherapy*	
Yes	105 (86.1%)
No	17 (13.9%)

*Chemotherapy approach*	
S-1	36 (29.5%)
CapeOX/SOX	69 (56.6%)

*Lauren type*	
I (intestinal type)	108 (88.5%)
D (diffuse type)	7 (5.7%)
M (mixed type)	7 (5.7%)

*Tumor deposit*	
Yes	21 (17.2%)
No	101 (82.3%)

Abbreviations: CapeOX-capecitabine and oxaliplatin, SOX-S-1 plus oxaliplatin, AD-adenocarcinoma.

**Table 2:** Univariate analyses of risk factors for lymph node metastasis.

Variable	Nodal-positive group	Nodal-negative group	*χ*2	*p* value
*Gender*			0.301	0.583
Male	67	31		
Female	15	9		

*Age (years)*			0.084	0.772
≤60	14	6		
>60	68	34		

*Tumor size*			7.342	0.007
≤4 cm	36	28		
>4 cm	46	12		

*Depth of invasion*			22.987	<0.001
PT1	2	13		
PT2	6	3		
PT3	41	16		
PT4a/PT4b	33	8		

*Histologic classification*			0.606	0.436
Moderately differentiated	41	17		
Moderately poorly differentiated	32	22		
Poorly differentiated	9	1		

*Histology*			2.173	0.140
Conventional AD	65	36		
Mucinous AD	7	3		
Papillary AD	4	0		
Signet-ring cell carcinoma	6	0		
Adenosquamous carcinoma	0	1		

*Tumor location*			3.334	0.343
Upper 1/3	27	14		
Middle 1/3	5	5		
Lower 1/3	39	19		
Mix	11	2		

*Vascular invasion*			31.991	<0.001
(−)	25	34		
(+)	57	6		

*Neural invasion*			13.922	<0.001
(−)	32	30		
(+)	50	10		

*Lauren type*			1.600	0.206
I (intestinal type)	70	38		
D (diffuse type)	5	2		
M (mixed type)	7	0		

*Tumor deposit*			3.940	0.047
Yes	18	3		
No	64	37		

Abbreviations: AD-adenocarcinoma.

**Table 3:** Multivariate analyses of risk factors for lymph node metastasis.

Variables	Hazard ratio (95% CI)	*p*-value
Tumor size (≤4 cm/>4 cm)	6.213 (2.097–18.407)	0.001
Neural invasion (No/Yes)	2.876 (1.011–8.184)	0.048
Vascular invasion (No/Yes)	16.881 (5.207–54.727)	<0.001
Tumor deposit (No/Yes)	3.147 (0.543–18.235)	0.201
AJCC T stage (T1−3/t4)	0.800 (0.221–2.898)	0.734

**Table 4:** Cox regression analysis of prognostic factors for overall survival.

Variable	Univariate analyses				Multivariable analyses	
	Number (*n*)	3-OS (%)	*χ*2	*p* value	Hazard ratio (95% CI)	*p* value
*Age (years)*			0.031	0.861		
≤60	20	54.5				
>60	102	59.1			

*Gender*			1.404	0.236		
Male	98	61.7				
Female	24	44.7				

*Tumor size (cm)*			3.699	0.054		
≤4 cm	64	66.3				
>4 cm	58	49.7				

*Depth of invasion*			35.969	<0.001	4.615 (2.182–9.759)	<0.001
PT1-3	81	79.8				
PT4	41	7.8				

*Lymph node metastasis*			17.360	<0.001	2.718 (0.863–8.564)	0.088
No	40	86.0				
Yes	82	44.0				

*Histologic classification*			2.003	0.367		
Middle-differentiated	58	64.2				
Middle-poor differentiated	54	55.5				
Poor-differentiated	10	45.0				
Histology			0.061	0.805		
Conventional AD	101	60.6				
Other	21	44.0				

*Type of gastrectomy*			0.418	0.811		
Proximal subtotal gastrectomy	20	48.3				
Distal gastrectomy	28	56.4				
Total gastrectomy	44	65.5				

*Type of surgery*			0.170	0.680		
Open surgery	56	60.7				
Laparoscopic surgery	66	55.7				

*Neural invasion*			18.978	<0.001	1.566 (0.732–3.354)	0.248
(−)	62	75.8				
(+)	60	39.7				

*Vascular invasion*			28.518	<0.001	3.036 (1.369–6.736)	0.006
(−)	59	78.8				
(+)	63	35.3				

*Lauren type*			8.825	0.003	2.175 (0.963–4.915)	0.062
I (intestinal type)	108	61.3				
D-M (diffuse-mix type)	14	41.7				

*Tumor deposit*			7.847	0.005	0.849 (0.413–1.747)	0.849
Yes	21	13.5				
No	101	66.5				

Subgroup analysis (AJCC stage II–IV)				

*Chemotherapy approach*			3.511	0.061		
S-1	36	39.3				
CapeOX/SOX	69	56.3				

## Data Availability

All the data used to support the findings of this study are included in the article.
